# Pennsylvania Medical College

**Published:** 1852-07

**Authors:** 


*Pennsylvania Medical College.*—The following notice of new appointments in the above mentioned institution, is copied from the Med. Examiner:

We learn, with much pleasure, that the faculty of this institution is about to be strengthened by the accession of Drs. F. G. Smith, J. M. Allen, and J. J. Reese, who have accepted the Professorships of *Institutes of Medicine*, *Anatomy*, and *Medical Chemistry and Pharmacy.* The two last named chairs have been lately vacated by death and resignation; the chair of Institutes is a new creation. These appointments are in every respect excellent, and cannot fail to advance the interests of the school in question. Drs. Smith and Allen have for many years filled lectureships in the Philadelphia Association for Medical Instruction, on the branches to which they are now translated in the Pennsylvania College; and the reputation which they have established, as eloquent, erudite, and judicious teachers, is second to none in the country. Dr. Reese has been for some time connected with the Philadelphia Medical Institute as a lecturer on Materia Medica and Therapeutics, and has been most favorably distinguished, both as a teacher and as a writer on this and other branches of medicine. His success in the chair of Materia Medica is a guarantee of his ability to do full justice to the kindred subject which he now undertakes.

We may also say, in noticing these appointments, that the high personal character and professional standing of the gentlemen named, cannot but give strength and popularity to any school with which they are connected. The Pennsylvania Medical College has already attained a most respectable position, and under this new organization, may rapidly anticipate a brilliant career. We are sure that it will have the cordial good wishes of the entire profession in Philadelphia. B.

---

*Spongio Piline.*—The new article called *spongio piline*, seems admirably adapted to the purposes for which it has been prepared. It is composed of a layer of sponge a third or a half an inch in thickness attached to india rubber cloth. Soaked in hot or warm water it is designed to serve as a poultice, or a fomentation, and it may also be used as a vehicle for medicated lotions. For each of these objects it will be found very convenient, and useful. A. I. Mathews, of this city, is supplied with the article.

